# The Evaluation of Ammonium Sulphate as a Potential Draw Solute in a Hybrid FO-RO Process to Concentrate Nutrients (NPK) from a Simulated Liquid Digestate—Part I: Deionized Water as a Feed Solution

**DOI:** 10.3390/membranes15120366

**Published:** 2025-12-01

**Authors:** Marsa Tolouei, Roshan Abraham, Niloofar Abdehagh, Majid Sartaj, Boguslaw Kruczek

**Affiliations:** 1 Department of Chemical and Biological Engineering, University of Ottawa, 161 Louis Pasteur, Ottawa, ON K1N 6N6, Canada; ztolo031@uottawa.ca (M.T.); rabra072@uottawa.ca (R.A.); 2 Natural Resources Canada (NRCan), 580 Booth Street, Ottawa, ON K1A 0E4, Canada; nabdehag@uottawa.ca; 3 Department of Civil Engineering, University of Ottawa, 161 Louis Pasteur, Ottawa, ON K1N 6N6, Canada; msartaj@uottawa.ca

**Keywords:** nutrient recovery, forward osmosis, reverse osmosis, hybrid FO-RO process, draw solution, ammonium sulfate

## Abstract

The ultimate objective of this research is to concentrate nutrients—nitrogen (N), phosphorus (P), and potassium (K)—and produce process water from a chemically pretreated liquid digestate using an FO-RO hybrid process. However, in this manuscript, we assessed the suitability of (NH_4_)_2_SO_4_ and NaCl as draw solutes in a series of FO experiments employing a commercial CTA membrane and DI water as the feed solution. We also examined the regeneration of (NH_4_)_2_SO_4_ in a series of RO experiments at various feed concentrations and pressures using a commercial polyamide (PA) thin-film composite (TFC) membrane, ACM4. Additionally, the RO experiments enabled the experimental determination of the osmotic pressure of (NH_4_)_2_SO_4_ at various feed concentrations, which is crucial for designing the FO part of the hybrid process. The CTA membrane exhibited a significantly greater selectivity for (NH_4_)_2_SO_4_ than for NaCl at any osmotic pressure. The RO experiments demonstrated the possibility of reconcentrating (NH_4_)_2_SO_4_ to 0.5 mol/L, with a corresponding water flux of 60 L h^−1^ m^−2^ at 40 bars. The experimentally determined osmotic pressures were lower than those predicted by van’t Hoff’s equation but were consistent with those reported in the literature using an indirect hygrometric method.

## 1. Introduction

Anaerobic digestion (AD) is a viable process for breaking down a variety of biodegradable organic matter, resulting in two main products: biogas and a nutrient-rich liquid, known as digestate [1,2]. Liquid digestate is a potential biofertilizer that consists of nitrogen (N) in the form of ammonia, phosphorus (P) in the form of orthophosphate, and potassium (K) in the form of potassium oxide, along with traces of metals and micronutrients [3]. The composition and solid content of the liquid digestate depend on the type of feedstock used in the AD process [4]. The common feedstock includes animal manure, food and agricultural wastes, and municipal wastewater sludge [5]. Due to its large volume, the direct application of digestate as a fertilizer is constrained by a low nutrient-to-volume ratio [5]. The large volume of digestate also increases transportation and storage costs. Although digested can be stored in open lagoons during non-winter periods, this practice contributes to greenhouse gas emissions [6,7].

The problem of a low nutrient-to-volume ratio in digestate is addressed using various nutrient recovery methods, including struvite precipitation and chemical treatment, such as coagulation/flocculation processes [8,9,10,11,12]. In struvite precipitation, specific nutrients are selectively separated from the digestate in solid, crystallized struvite precipitants [13]. In chemical treatment, metal salts, such as ferric chloride and aluminum sulfate, react with dissolved phosphorus to form soluble phosphates in the form of solid flocs [14]. In both methods, the nutrient compounds are removed as solids, and as such, they are not readily accessible to crops [15]. In another approach, mechanical separation methods, such as centrifuges, filter presses, and screw presses, are applied to remove solids from the digestate, reducing the volume to facilitate handling and transportation of the remaining liquid. The removed solids represent solid biofertilizers [16]. This approach does not allow for the selective capture of specific nutrients. Therefore, mechanical separations are typically used as pretreatment and are often combined with other methods [17].

Membrane separation technologies, particularly pressure-driven processes such as reverse osmosis (RO) and nanofiltration (NF), represent a unique category of mechanical separation methods commonly used in wastewater treatment [18,19]. For example, Niewersch et al. (2014) utilized NF to concentrate phosphoric acid and potassium from sewage sludge, reporting recoveries of 50% and 30%, respectively [20]. In another study, Blöcher et al. (2012) reported a 54% recovery of phosphoric acid by the NF process, in which the wastewater feed was oxidized before being sent to the membrane unit [21]. Adam et al. (2018) combined NF with a screw press as a pretreatment method to reduce the solid content in the feed entering the NF unit [22]. The permeate from the NF process was sent to an RO unit to increase the recovery of nutrients. They reported a recovery rate of more than 90% for phosphorus from the feed digestate. However, the corresponding recoveries of ammonium and potassium were low, while membrane fouling was significant, particularly in the NF process [22].

In recent years, forward osmosis (FO) has been considered as a dewatering step in wastewater treatment, concentrating nutrients in liquid digestate [23,24,25,26]. FO utilizes the osmotic pressure difference of solutions across a semipermeable membrane to draw water from a dilute feed solution (FS) to a more concentrated draw solution (DS) [27,28]. As a result, solutes in the FS are concentrated while the DS is diluted [29,30]. Unlike pressure-driven membrane processes such as NF and RO, FO does not require hydraulic pressure, reducing the membranes’ operational costs and fouling propensity [31]. However, unless a dilute DS is a product, such as dilute seawater for water desalination in a low-pressure RO process rather than a high-pressure RO process, the DS must be reconcentrated in a subsequent NF or RO process [32,33]. In other words, a pressure-driven membrane process is still required. However, the feed solution of the subsequent NF or RO process is a “clean” diluted DS rather than a “dirty” liquid digestate. Holloway et al. used a bench-scale FO system, using NaCl as the draw solute, to concentrate ammonia, organic nitrogen, and orthophosphate in liquid digestate (FS) from biomass with and without pretreatment. They reported up to 75% volume reduction of the pretreated liquid digestate when using a 70 g/L NaCl DS, and the diluted DS was reconcentrated in the subsequent RO process [26].

Sodium chloride, because of its high osmotic pressure, is widely used as a draw solute in FO processes. At the same time, because NaCl is a monovalent salt with a small ionic radius, it can easily diffuse into the feed solution, thereby decreasing the osmotic pressure driving force [34]. Also, the high osmotic pressure of NaCl implies that its recovery requires high hydraulic pressure and is limited to RO processes [35,36]. Consequently, other draw solutes were considered for FO processes to recover nutrients. For example, Hau et al. used ethylenediaminetetraacetic acid (EDTA) disodium salt as a draw solute for nutrient recovery from sewage sludge in a hybrid FO-NF process using commercial asymmetric cellulose triacetate in the FO process. They reported a water flux of 8.45 L/m^2^/h when using 0.7 mol/L sodium EDTA as the DS during the first hour of operation at pH 8. Also, in the FO process, the rejection of the nutrient compounds (NH_4_^+^-N and PO_4_^3−^-P) from the feed sludge solution were 97% and 99%, respectively. Moreover, the DS was reconcentrated using an NF membrane (TS80) rather than an RO membrane, resulting in a DS rejection of 93% [37]. Ammonium sulphate, (NH_4_)_2_SO_4_, offers relatively high osmotic pressure, and because it is a multivalent salt, it should minimize the undesirable reverse solute flux in the FO process. Consequently, (NH_4_)_2_SO_4_ has garnered significant interest as a draw solute in fertilizer-drawn forward osmosis (FDFO) for dewatering wastewater [38,39] and concentrating dilute brine solutions [40,41]. However, unlike other draw solutes such as Na_2_SO_4_, MgCl_2_, NH_4_HCO_3_, and diammonium phosphate, ammonium sulphate has not been explored in hybrid FO-RO processes, where, in addition to dewatering various feed solutions, the draw solute is recovered during the subsequent RO process. Therefore, this study aims to address this research gap by utilizing (NH_4_)_2_SO_4_ as a draw solute in an FO–RO hybrid system with the respective commercial CTA and TFC membranes under digestate-compatible concentration conditions.

The ultimate goal of this research is to recover NPK nutrients using an FO-RO hybrid process from a simulated chemically pretreated liquid digestate. However, in this paper, which represents the first phase of this research, we evaluate the suitability of (NH_4_)_2_SO_4_ as a draw solute in a series of FO experiments employing a commercial CTA membrane and DI water as the feed solution and compare its performance with that of NaCl as a benchmark. In addition to FO performance, we also examine the regeneration of (NH_4_)_2_SO_4_ in a series of RO experiments at various feed concentrations and pressures using a commercial polyamide (PA) thin-film composite (TFC) membrane, ACM4. Furthermore, the RO experiments were designed to determine the exact osmotic pressure of (NH_4_)_2_SO_4_ at different feed concentrations. These experimental osmotic pressures are essential for designing the FO component of the hybrid process to recover NPK nutrients.

## 2. Theoretical Background

### 2.1. Reverse Osmosis

According to the SD model, the rate equations for the water (*J_w_*) and solute (*J_s_*) fluxes in RO membranes are given by:

(1) $$J_{w}=A\left(\Delta p-\Delta \pi \right)$$

(2) $$J_{s}=B\Delta c$$

where A and B are the water and solute permeability coefficients, Δ*p* and Δ*π* are the hydraulic and osmotic pressure gradients across the membrane, and Δ*c* is the molar concentration gradient. In reality, A and B are not permeabilities but rather permeances. However, we will use the term permeability to be consistent with the literature. As a first approximation, the osmotic pressure is directly proportional to the molar concentration of solute and is evaluated using van’t Hoff’s equation:

(3) π=icRT

where *i* is the number of ions the solute dissociates, *R* is the universal gas constant, and *T* is the absolute temperature.

In the absence of external concentration polarization (ECP), the concentration gradient across the membrane is:

(4) $$\Delta c=c_{f}-c_{p}$$

where *c_f_* and *c_p_* are the molar solute concentrations in the bulk feed and permeate. If the ECP occurs, the concentration of solute in contact with the membrane at the feed (*c*_*f*,*m*_) is greater than *c_f_*, which increases the driving force for *J_s_*. At the same time, an increase in Δ*c* increases Δ*π*, thus reducing the driving force for *J_w_*. Although the ECP may also occur at the permeate side of the membrane, because of the magnitude of *c_p_*, which often approaches zero, its effects are negligible. The magnitude of the concentration polarization is expressed by the ratio, *c*_*f*,*m*_/*c_p_*, which is referred to as the concentration polarization modulus.

The selective properties of RO membranes are expressed by the solute rejection (R). In the absence of the ECP:

(5) $$R=1-\frac{c_{p}}{c_{f}}$$

If the ECP occurs, the R from Equation (5) represents an observed solute rejection, which, because *c_f_* < *c*_*f*,*m*_, underestimates the intrinsic solute rejection (R′). When the EPC is negligible, R = R′. In that case, the solute rejection is related to the water and solute permeabilities by:

(6) $$R=\frac{A\left(\Delta p-\Delta \pi \right)}{A\left(\Delta p-\Delta \pi \right)+B}$$

Knowing A and Δ*π* (Δ*p* is a controlled parameter) enables the determination of B, which is an intrinsic membrane property for a given solute and the process temperature. Unlike B, R is not constant; it depends on the applied hydraulic pressure and the feed concentration at a given temperature.

### 2.2. Forward Osmosis

Unlike pressure-driven membrane processes, forward osmosis operates without external hydraulic pressure. Therefore, according to the SD model, the water flux given by Equation (1) becomes:

(7) $$J_{w}=-A\Delta \pi$$

The solute flux in FO is given by Equation (2), but the meaning of Δ*c* changes to:

(8) $$\Delta c=c_{d}-c_{f}$$

where *c_d_* and *c_f_* refer to the molar concentrations of the bulk, draw and feed solutions. It follows that in FO, *c_d_* > *c_f_*, and consequently, the osmotic pressure gradient in Equation (7) is given by:

(9) $$\Delta \pi =\pi_{d}-\pi_{f}$$

The negative sign in Equation (7) indicates that the solvent and solute move in opposite directions. Consequently, *J_s_* in FO is often referred to as a reverse solute flux to signify that.

In principle, solute rejection in FO would be given by:

(10) $$R=1-\frac{c_{f}}{c_{d}}$$

However, the selectivity (S) of FO membranes is not expressed in terms of R but rather by the ratio of *J_w_* and *J_s_*, i.e.,

(11) $$S=\frac{J_{w}}{J_{s}}$$

Like RO and other pressure-driven membrane separation processes, FO is susceptible to ECP. However, because there is no applied hydraulic pressure, the ECP in FO processes is much less severe than in RO [37]. On the other hand, the opposite directions of the water and solute transport give rise to an inherent internal concentration polarization (IPC) in FO. The ICP occurs within the pores of the membrane support layer and cannot be eliminated or reduced by creating turbulence near the membrane surface, as in the case of EPC. The ICP can be minimized by increasing the support layer porosity and/or decreasing its tortuosity, thereby reducing the resistance to solute diffusion through the support layer. In other words, minimizing ICP requires structural modifications to the FO membrane. The ICP always leads to a decrease in Δ*π* in Equation (7), the driving force for *J_w_*.

The FO membranes can be operated in two orientations: the active layer-facing draw solution (AL-DS) and the active layer-facing feed solution (AL-FS). In the AL-DS orientation, *π*_*f*,*m*_ > *π_f_* and the resulting concentration polarization is called a concentrative ICP. On the other hand, in the AL-FS orientation, *π*_*d*,*m*_ < *π_d_* and the resulting concentration polarization is referred to as dilutive ICP.

### 2.3. Osmotic Pressure

The van’t Hoff equation, Equation (3), used for the preliminary evaluation of the osmotic pressure gradients in the RO and FO processes, applies to ideal solutions. More specifically, it assumes complete dissociation of the solute, no interactions between dissociated ions, and that their cumulative volume is negligible, i.e., the partial molar volume equals the solution volume. These assumptions might apply to infinitely dilute solutions. However, as the solute concentration increases, they fail. Morse and Frazer suggested using a molal rather than molar solution concentration (i.e., moles per mass rather than moles per solvent volume) in the van’t Hoff equation [42]. However, compared to the conventional van’t Hoff equation, this approach deviates only for relatively high-molecular-weight solutes, and it does not accurately predict osmotic pressure for most solutions at high concentrations [43]. The deviation from Equation (3) at higher solute concentrations can be positive or negative, and van’t Hoff equation is modified to:

(12) π=ϕicRT

where *ϕ* is a dimensionless osmotic coefficient, greater or smaller than unity. Moreover, *ϕ* is not constant and, for a given temperature, varies with the solute concentration [44,45].

Osmotic pressure is an example of a colligative property that connects it to other properties, such as vapour pressure, boiling point, and freezing point. In other words, the presence of dissolved solute in the solution, which generates the osmotic pressure, also affects the solution’s vapour pressure (a decrease), boiling point (an increase) and freezing point (a decrease) compared to the pure solvent. Changes in these properties, such as osmotic pressure, are related to the solution’s concentration. Monitoring these properties as a function of solute concentration allows the experimental determination of the osmotic pressure [46].

Osmotic pressure can also be determined directly using membrane osmometry. Osmometry measurements are typically performed in static cells and rely on achieving thermodynamic equilibrium [46,47,48,49]. Static cells typically contain two chambers separated by a semipermeable membrane. One chamber contains the tested solution, and the other the pure solvent. Due to the osmotic pressure, the solvent flows into the solution container until equilibrium is reached. The resulting equilibrium pressure difference is the solution’s osmotic pressure. However, due to solvent transport, the equilibrium concentration in the solution chamber is lower than the initial concentration, and it can take a considerable amount of time to reach equilibrium in a static system.

Alternatively, RO testing systems can measure the osmotic pressure directly in dynamic experiments. If an RO membrane can completely or nearly completely reject solute, according to Equation (1), performing experiments with a given feed concentration at different hydraulic pressures should yield a linear relation between *J_w_* and Δ*p*. If such a linear relationship exists, the slope represents water permeability (A), and the intercept at *J_w_* = 0 is Δ*π*. Moreover, if solute rejection occurs, R ⟶ 1, i.e., *c_p_* ≈ 0, then Δ*π* ≈ *π_f_*. This method, first proposed by Merson and Morgan [50], was adopted in the current work. The significant advantages of this method include confidence in the solute concentration, represented by *c_f_*, at which the osmotic pressure is determined, and achieving a steady-state permeation in a fraction of the time required to reach equilibrium. On the other hand, this method requires a large volume of solutions. However, in this project, since we aimed to regenerate the draw solution in RO experiments, the large volume of feed solution was not a concern. It is important to note that to overcome the challenge of a large volume of tested samples, Nabetani et al. developed an osmometer to conduct RO experiments in a dead-end filtration cell [46]. However, the experiments in a dead-end filtration cell are inherently transient, and *c_f_* continuously changes as the permeate is collected [43].

## 3. Materials and Methods

### 3.1. Materials

The FO membrane used in this study was a commercial cellulose triacetate (CTA) membrane purchased from FTSH2 Industries, Inc., Albany, OR, USA. According to the supplier, the CTA membrane can be operated at a maximum temperature of 50 °C, a minimum transmembrane pressure of 5 psi, and a maximum inlet pressure of 75 psi. The RO membrane was a commercial thin film composite (TFC) membrane purchased from TriSep Industries, Inc., Goleta, CA, USA. The TFC membrane consisted of an active polyamide (PA) layer supported by a polysulfone (PS) layer and a polyethylene terephthalate (PET) non-woven fibre layer. According to the manufacturer, this low-pressure, high-flux membrane has a rejection of NaCl of 99.2% and a water flux of 30/150 gfd/psi. Ammonium sulfate, used to prepare the draw solution in the FO experiments and the feed solution in the RO experiments, had a purity greater than 98.0% and was purchased from Thermo Fisher Scientific, Waltham, MA, USA. Sodium chloride, used as a reference draw solute in the FO experiments, had a purity greater than 99.0% and was obtained from Thermo Fisher Scientific, Waltham, MA, USA. Deionized (DI) water was used as the feed solution in the FO experiments and to prepare all solutions for the FO and RO experiments. The DI water was also used to clean the FO and RO systems before and between experiments.

### 3.2. Methods

**Figure 1:**
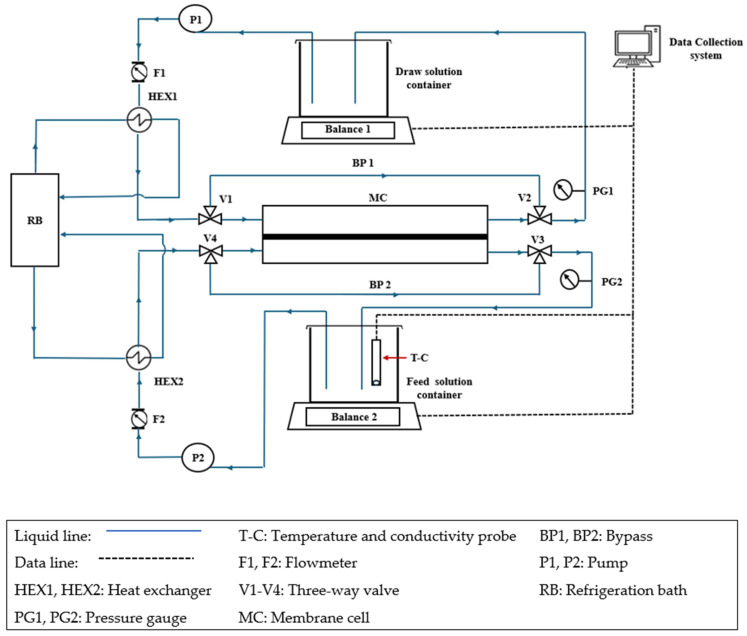
Schematic diagram of the FO testing setup. This is the modified version of the system reported in Ref. [51].

Figure 1 presents the schematic diagram of the FO system used in this study; it is a modified version of the FO system described by Du et al. [51]. The FO membrane was installed in a Teflon crossflow symmetric membrane cell (CF016D-FO Cell) purchased from Sterlitech, Auburn, WA, USA. The effective membrane area and hold-up volume were 20.6 cm^2^ and 4.7 cm^3^. Pumps P1 and P2, along with flow meters F1 and F2, controlled the circulation rates at the draw and feed sides of the membrane. Both sides were maintained at atmospheric pressure, as verified by the respective pressure gauges PG1 and PG2. The experiments were performed at 21.5 °C (±0.5 °C), with temperature control achieved using a refrigerated bath circulator and heat exchangers HEX1 and HEX2. Balance 1, Balance 2, and the conductivity/temperature meter (T-C) were connected to a personal computer, and the experimental data were recorded using a LabVIEW data acquisition program.

The FO membrane inside the cell was thoroughly washed by circulating DI water on both sides. Once the circulating water conductivity stabilized, the feed and draw solution tanks were filled with fresh deionized (DI) water. The membrane washing before and between tests continued until the conductivity of DI water (1.7–2.5 µs/cm) was achieved, which required several batches of DI water. Then, the draw solution tank was refilled with the desired draw solution type and concentration, and the system was switched to bypass mode. The draw and feed solution containers each held 1 L. However, the draw and feed solution volumes were 500 mL and 700 mL, respectively. These volumes were selected to maintain a relatively constant, reasonable resolution for measuring the feed solution’s conductivity during 30-minute experiments. The circulation rates on both sides were 1.6 L/min. While in bypass mode, the membrane inside the cell was in contact with DI water on both sides. The system remained in bypass mode until the set temperature was reached and stabilized. The experiment was initiated by redirecting the draw and feed solutions from the respective bypass lines to the draw and feed sides of the membrane cell, a process that took less than 2 s.

The FO experiments used (NH_4_)_2_SO_4_ as the draw solute at six different molar concentrations: 0.1, 0.25, 0.35, 0.5, 1.0, and 2.0 mol/L. The FO performance of (NH_4_)_2_SO_4_ was compared to that of NaCl, a commonly used draw solute. The concentrations of the NaCl draw solutions were chosen to match the theoretical osmotic pressures of the (NH_4_)_2_SO_4_ draw solutions at 0.1, 0.25, 0.35, and 0.5 mol/L. In all FO experiments, DI water served as the feed solution. All FO experiments were performed with the active-layer-facing feed-solution (AL-FS) orientation of the CTA membrane.

**Figure 2:**
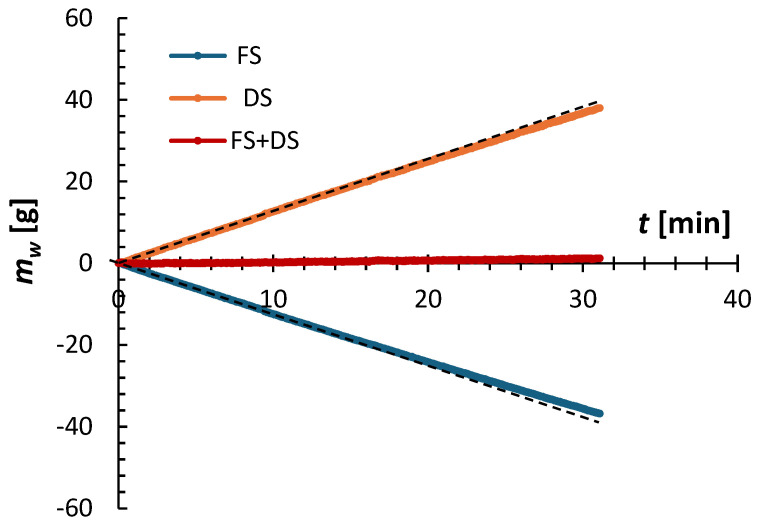
Example of the progress of an FO experiment based on the mass of the feed and draw solutions as a function of time. The experiment was performed using a CTA membrane, operated in the AL-FS orientation, with 2 M (NH_4_)_2_SO_4_ as the draw solution and DI water as the feed solution at *T* = 21.5 ± 0.5 °C.

Figure 2 shows an example of the FO experiment’s progress, illustrating the normalized mass of the feed and draw solutions, along with the total mass, over time. Details of processing raw experimental data are provided elsewhere [51]. It is clear that immediately after starting the experiment, the masses of the feed and draw solutions become linear functions of time. Additionally, the change in the feed solution’s mass mirrors the change in the draw solution’s mass relative to the time axis. Consequently, the combined mass of the feed and draw solutions remains constant, confirming the experiment’s validity.

The experimental water flux was calculated from:

(13) $$J_{w}=\frac{dm_{w}/dt}{\rho A_{m}}$$

where *ρ* is the density of water, and *A_m_* is the membrane area. $dm_{w}/dt$ was determined from the linear regression of the first 20 min of the experimental mass of the feed solution (or draw solution), which is represented by the black dashed line. Beyond the first 20 min, the rate of mass change decreased slightly due to dilution of the draw solution. As shown in Figure 2, during a 30 min experiment, approximately 40 g of water permeated into the draw solution, representing 5.7% of its initial mass. This is why *J_w_* calculated using Equation (13) can be considered a steady-state value.

**Figure 3:**
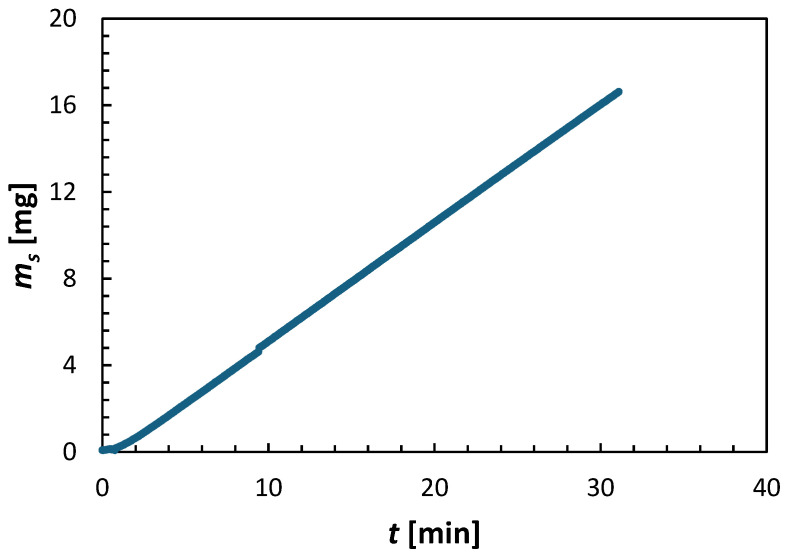
Example of the progress of an FO experiment based on the mass of the draw solute in the feed solution over time. The experiment was conducted using a CTA membrane, operated in the AL-FS orientation, with 2 M (NH_4_)_2_SO_4_ draw solution and DI water feed at *T* = 21.5 ± 0.5 °C.

Figure 3 presents the increase in the mass of the draw solute in the feed solution in the experiment depicted in Figure 2. The mass of the draw solute (*m_s_*) in the feed solution was calculated from:

(14) $$m_{s}=c_{s}V_{FS}$$

where *c_s_* and *V_FS_* represent the concentration of the draw solute in the feed solution and the volume of the feed solution, respectively. *c_s_* was determined from the feed solution’s measured conductivity, while *V_FS_* was calculated from its measured mass. Both *c_s_* and *V_FS_* continuously varied throughout the experiment. There is a slight delay (about 30 s) before the mass of the draw solute in the feed solution appears. This may suggest a dynamic in the transport of the solute from the draw to the feed side. However, after this brief delay, the *m_s_* increases linearly with time, like the rate of change in the mass of the feed and draw solutions in Figure 2. Consequently, the reverse solute flux was calculated using the linear regression of the first 20 min of the experiment (excluding the initial delay), shown in Figure 3.

(15) $$J_{s}=\frac{dm_{s}/dt}{A_{m}}$$

**Figure 4:**
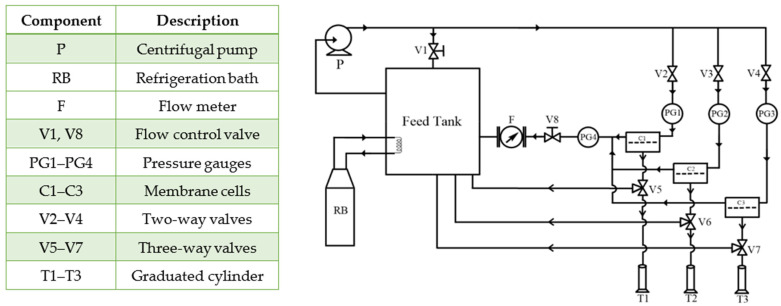
Schematic diagram of the RO testing system.

To evaluate regeneration of the (NH_4_)_2_SO_4_ draw solution in the RO process, experiments were performed in a crossflow system, as shown in Figure 4. The RO system used in this work is an updated version of the one described by Carter et al. [52]. More specifically, the feed flow rate and feed pressure were decoupled by introducing a bypass line equipped with a flow control valve, V1. With V1 open and the two-way valves V2-V4 closed, the system operated entirely in bypass mode. The bypass was used before the experiments to allow the system to reach the set temperature of 21.5 °C ± 0.5 °C. Additionally, the bypass line facilitated the mixing of the contents in the feed tanks. With V2-V4 open, the feed flow was split between the bypass and the permeation cell. The other flow control valve, V8, controlled the feed pressure. Gradually closing the V8 valve increased the feed pressure while decreasing the feed flow rate. A partial closing of V1 compensated for the resulting decrease in the feed flow rate. Maintaining a high feed flow rate regardless of the feed pressure was essential to minimize the effects of the ECP. The RO experiments were performed with concentrations of (NH_4_)_2_SO_4_ in the feed ranging from 0.1 to 0.5 mol/L at pressures ranging from 10 to 40 bar, while the feed flow rate was maintained at 3.5 L/min ± 0.5 L/min. The maximum feed concentration of 0.5 mol/L was established based on the theoretical osmotic pressure of this feed solution (Δ*π_t_* = 36.31 bar) and the maximum feed pressure (40 bar).

Three membrane coupons were tested simultaneously to increase confidence in RO results. However, any membrane cell could be isolated from the system using valves V2-V4 and V5-V7, while maintaining the constant feed pressure and flow rate by adjusting valves V1 and V8. The RO experiments were conducted for up to 3 h to ensure steady-state conditions. Permeate samples from each cell were collected simultaneously, approximately every 30 min. Specifically, using a stopwatch, the time (*t*) was recorded to collect at least 10 mL of permeate (*V*), and water flux was calculated from:

(16) $$J_{w}=\frac{\Delta V}{\Delta tA_{m}}$$

where *A_m_* is the membrane permeation area, which in each membrane cell was 12.56 cm^2^. The solute concentration in permeate samples was determined from their electrical conductivity. The electrical conductivity of the feed stream was also monitored in the feed tank.

**Figure 5:**
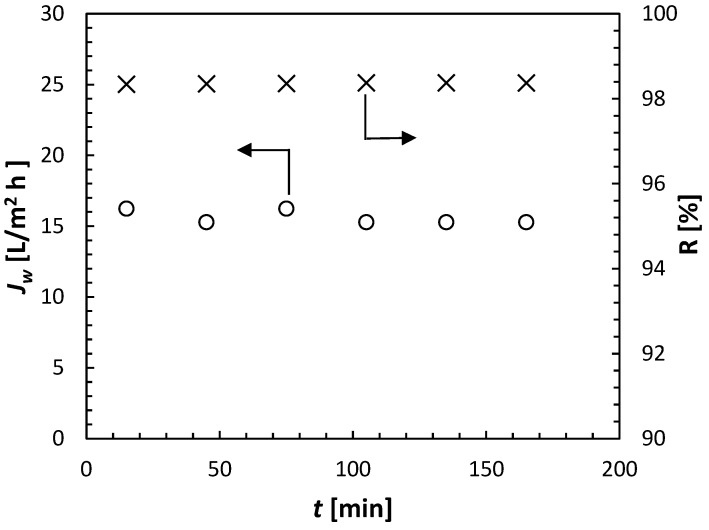
Sample of experimental results from cell C1 in the experiments using ammonium sulfate (NH_4_)_2_SO_4_ at 0.5 mol/L, with Δ*p* = 30 bar and *T* = 21.5 ± 0.5 °C.

When the permeate was not collected for analysis, it was automatically recycled back to the 20-litre feed tank. In each experiment, the volume of the feed solution was 14 litres. After measuring their conductivity, the collected permeate samples were returned to the feed tank, allowing the feed solution to maintain a constant conductivity (solute concentration) throughout the entire 3 h permeation tests. Figure 5 presents a sample of the experimental results, i.e., *J_w_* and R as a function of time. Solute rejection was calculated using Equation (5) based on the measured conductivities of the permeate and feed streams. It can be noticed that both *J_w_* and R remained practically constant during the entire experiment. The arithmetic average of the recorded *J_w_* and R will be reported in further analysis.

## 4. Results and Discussion

### 4.1. Forward Osmosis

The FO experiments can be conducted in two membrane orientations: the active-layer-facing-feed solution (AL-FS) and the active-layer-facing-draw solution (AL-DS). The latter orientation typically exhibits a very high initial water flux due to reduced dilutive internal concentration polarization (ICP) (Onoda et al., 2016) [53]. However, as steady state is reached, *J_w_* decreases but remains slightly higher than in the AL-FS orientation. Conversely, *J_s_* in the AL-DS configuration is much greater than in the AL-FS, resulting in higher *S* in the AL-FS compared to the AL-DS [53,54]. Therefore, all experiments in this study were performed with the AL-FS membrane orientation.

**Figure 6:**
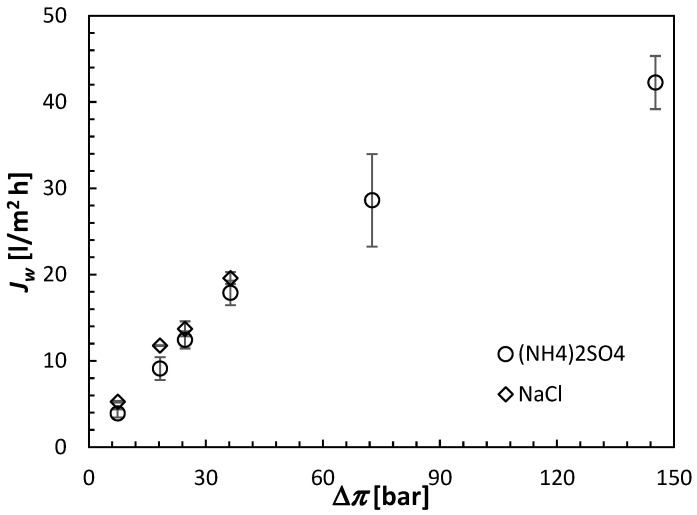
Water fluxes in the CTA membrane operated in the AL-FS orientation induced by the (NH_4_)_2_SO_4_ and NaCl draw solutions. The concentration of the draw solutions is represented by the theoretical osmotic pressure from Equation (3). All experiments were performed at 21.5 ± 0.5 °C using DI water as a feed solution.

**Figure 7:**
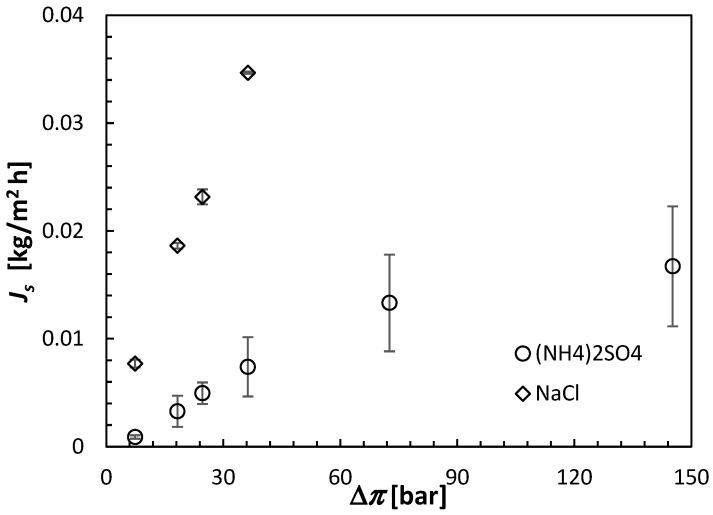
Reverse solute fluxes of (NH_4_)_2_SO_4_ and NaCl in the CTA membrane operated in the AL-FS orientation. The concentration of the draw solutions is represented by the theoretical osmotic pressure from Equation (3). All experiments were performed at 21.5 ± 0.5 °C using DI water as a feed solution.

**Figure 8:**
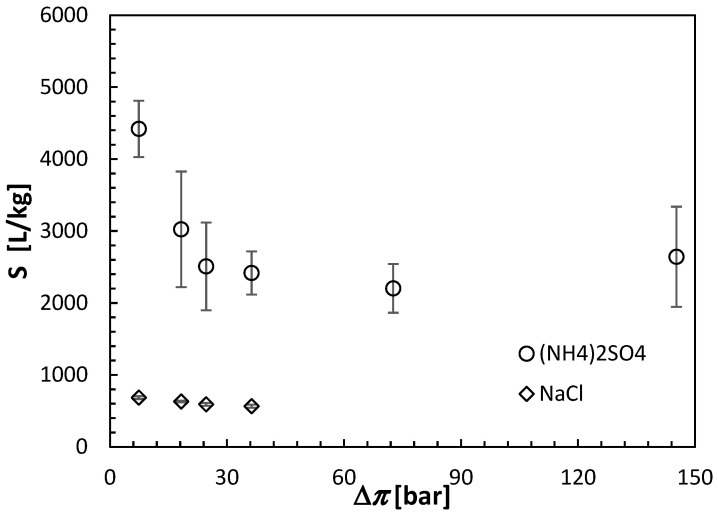
Selectivity of the CTA membrane in the AL-FS orientation in the FO experiments with ammonium sulfate and sodium chloride as draw solutes and DI water as a feed solution. The concentration of the draw solutions is represented by the theoretical osmotic pressures estimated using Equation (3). All experiments were performed at 21.5 ± 0.5 °C.

All FO results shown in this section are the averages of at least three separate CTA membranes from the same batch. Moreover, each membrane was carefully cleaned and retested under identical conditions after the initial test. The error bars in Figure 6, Figure 7 and Figure 8 indicate the corresponding standard deviations.

Figure 6 presents the relation between the water flux and the draw solution concentration for two draw solutes, (NH_4_)_2_SO_4_ and NaCl. NaCl is commonly used in the literature and can serve as a benchmark for (NH_4_)_2_SO_4_ performance. According to Equation (3), the same molar concentrations of NaCl and (NH_4_)_2_SO_4_ do not lead to the same osmotic pressures because of the different number of ions into which the two solutes dissociate. Consequently, the concentrations of the draw solutions in Figure 6 are expressed in terms of the theoretical osmotic pressures evaluated from Equation (3). It can be noticed that for similar theoretical osmotic pressures, *J_w_* induced by NaCl is greater than that induced by (NH_4_)_2_SO_4_. For example, for Δ*π* = 36.31 bar, the respective water fluxes are 19.61 L/m^2^ h compared to 16.81 L/m^2^ h. A similar difference between the water flux induced by NaCl (a monovalent draw solute) and a multivalent draw solute (MgCl_2_) was reported in the literature and was attributed to the complete dissociation of NaCl and the high mobility of the resulting Na^+^ and Cl^−^ ions [27].

The experiments with NaCl as a draw solute were performed up to the theoretical osmotic pressure of 36.61 bar. In that osmotic pressure range, *J_s_* appears to be directly proportional to the solute concentration, consistent with Equation (8). The same linear relation at relatively low concentrations is also evident for (NH_4_)_2_SO_4_. The slopes of *J_s_* versus Δ*π* in Figure 7 correspond to the respective permeabilities of the draw solutes. Consequently, according to Figure 7, B of NaCl in the CTA membrane is nearly 5 times greater than that of (NH_4_)_2_SO_4_. NaCl is a monovalent salt, and the resulting ions, Na^+^ and Cl^−^, have smaller hydration radii and greater mobility than NH_4_^+^ and SO_4_^−2^, which are the primary reasons for the much greater *J_s_* of NaCl compared to (NH_4_)_2_SO_4_. It is also important to note that at higher theoretical osmotic pressures, the rate of increase in *J_s_* with Δ*π* decreases, which is consistent with a decrease in *J_w_* observed in Figure 6. The driving force for *J_s_* is Δ*c* rather than Δ*π*.

It is important to note that although during the 30 min FO tests, *J_w_* with the NaCl draw solution was greater than that with the (NH_4_)_2_SO_4_ draw solution, because of the former’s much higher *J_s_*, the order of *J_w_* would likely change at longer times because of the faster dilution of the NaCl solution. However, in the current discussion, we will continue to focus on the 30 min FO experiments.

On the one hand, for a given theoretical osmotic pressure, *J_w_* was slightly greater, while the undesirable *J_s_* was much greater for NaCl than (NH_4_)_2_SO_4_. The assessment of the FO performance is conveniently evaluated based on the ratio of *J_w_* and *J_s_*, which can be considered as the selectivity (S) of the FO process, as expressed by Equation (11). Figure 8 combines Figure 6 and Figure 7 and compares the selectivity of the CTA membrane in the FO experiments using NaCl and (NH_4_)_2_SO_4_ as the respective draw solutes. It is evident that, although the selectivity of the CTA membrane in experiments with (NH_4_)_2_SO_4_ decreases significantly at low draw solute concentrations, it remains significantly greater than that for experiments with NaCl (2500–4700 L/kg versus 600–800 L/kg). For (NH_4_)_2_SO_4_, the best combination between *J_w_* and S is offered by the highest concentration of the draw solute of 2 mol/L. However, with this high concentration, the theoretical osmotic pressure is nearly 150 bar. Therefore, this concentration would be prohibitive for the reconcentration of the draw solution in the RO process. Consequently, based on the FO experiments presented in this section, it was established that a 0.5 mol/L concentration of (NH_4_)_2_SO_4_, corresponding to the theoretical Δ*π* of 36.61 bar, might be the optimum for future experiments with the actual feed solution, pending the confirmation of the possibility of regenerating the aqueous (NH_4_)_2_SO_4_ solutions to this concentration in the RO process.

### 4.2. Reverse Osmosis

The primary purpose of the RO experiments was to demonstrate the feasibility of regenerating the draw solution to its desired initial concentration of 0.5 mol/L of (NH_4_)_2_SO_4_. It required determining a minimum Δ*p* at which *J_w_* was observable at this feed solution concentration. According to Equation (3), the osmotic pressure gradient of the 0.5 mol/L aqueous solution of (NH_4_)_2_SO_4_ is 36.61 bar. Consequently, *J_w_* should be observed Δ*p* = 40 bar. However, given the shape of the *J_w_* curve for (NH_4_)_2_SO_4_ in Figure 6, it was speculated that Equation (3) might overestimate the osmotic pressure for this solute. Consequently, another objective of the RO experiments was to experimentally determine the osmotic pressure of (NH_4_)_2_SO_4_ for the contractions up to 0.5 mol/L used in the FO experiments.

All RO results presented in this section are the averages from three commercial TriSep TFC membrane coupons tested simultaneously in membrane cells C1-C3 (see Figure 4), and the error bars represent the respective standard deviations. Because the selectivity of the CTA membrane was significantly higher for (NH_4_)_2_SO_4_ than for NaCl, the RO experiments were performed only with feed solutions containing different concentrations of the former solute.

**Figure 9:**
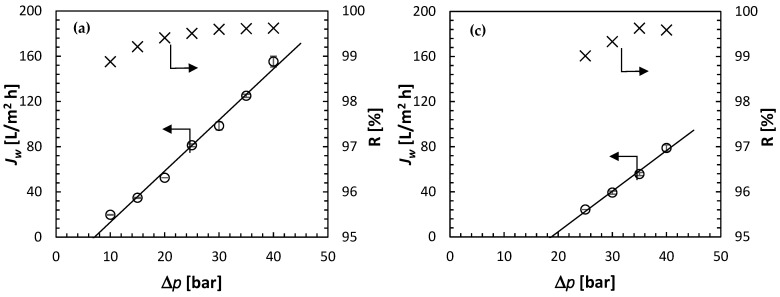

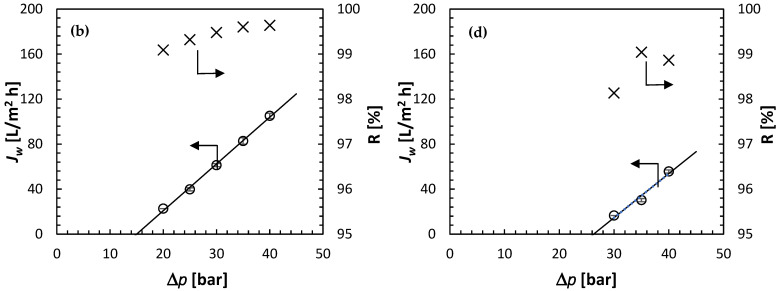
Summary of the RO experiments: water flux and solute rejection as a function of the applied pressure at different feed solution concentrations (0.1 mol/L—(**a**), 0.25 mol/L—(**b**), 0.35 mol/L—(**c**), 0.5 mol/L—(**d**)). Solute: (NH_4_)_2_SO_4_; all experiments at 21.5 ± 0.5 °C.

Figure 9 presents the summary of the RO experiments—*J_w_* and R as a function of Δ*p*—at four concentrations of the feed solution: 0.1 mol/L (Figure 9a), 0.25 mol/L (Figure 9b), 0.35 mol/L (Figure 9c) and 0.5 mol/L (Figure 9d). As expected based on Figure 6, for the highest feed solution concentration of 0.5 mol/L, the water flux was observed at feed pressures lower than the theoretical Δ*π* = 36.61 bar. Moreover, *J_w_* appears to increase linearly with Δ*p* and extrapolating the linear regression to *J_w_* = 0 leads to Δ*p* = 26.26 bar. The latter represents the experimental osmotic pressure. At the same time, it is evident in Figure 9d that the observed R, regardless of Δ*p* is greater than 98%. In other words, the concentration of solute in the permeate is much smaller than in the feed solution (i.e., *c_p_* << *c_f_*). Therefore, Δ*π ≈ π_f_* = 26.26 bar at *c_f_* = 0.5 mol/L.

For all feed concentrations, not only for *c_f_* = 0.5 mol/L, *J_w_* increases linearly with Δ*p*. Therefore, there is no evidence of membrane compaction, i.e., a decrease in the rate of the increase in *J_w_* with Δ*p* at higher feed pressures. For a given feed solution concentration, the solute rejection generally increases with Δ*p*, which is qualitatively consistent with the solution-diffusion model. According to Equation (1), the corresponding slopes in Figure 9 represent the water permeability coefficient, and the intercept at *J_w_* = 0 represents the osmotic pressure. Table 1 summarizes the experimentally determined A and *π* at different feed concentrations. The latter is compared to the theoretical *π* calculated from Equation (3), and the ratio of the two represents the osmotic coefficient in Equation (12). Knowing A, R, Δ*p* and Δ*π*, rearranging Equation (6) allows evaluation of the solute permeability in the membrane:

(17) $$B=\frac{A\left(\Delta p-\Delta \pi \right)\left(1-R\right)}{R}$$

where Δ*π* is approximated by the experimental *π*. The average values of B for each solute concentration are also included in Table 1.

According to the solution-diffusion model, A and B should be independent of feed concentration and pressure. Moreover, A should be independent of the solute in the feed solution. Consequently, the values of A in Table 1 for (NH_4_)_2_SO_4_ should be comparable to the manufacturer’s specification for the NaCl solution, which, for the solution with an unknown concentration, are R = 99.2% and *J_w_* = 30 gal/ft^2^ day at Δ*p* = 150 psi. These correspond to A = 4.92 L/m^2^ h bar, which is slightly greater than the A values in Table 1. The experimental water permeability generally decreases with an increase in the feed concentration. The exception is A at *c_f_* = 0.5 mol/L, slightly greater than A at *c_f_* = 0.35 mol/L. A decrease in A with *c_f_* might be due to more significant effects of the external concentration polarization at higher feed concentrations. Increasing the concentration of polarization modules would decrease the driving force for *J*_*w*,_ ultimately leading to the underestimation of A.

Considering solute rejection, as previously mentioned, it increases with Δ*p* reaching R as high as 99.64%, which is greater than the manufacturer-specified R = 99.2 for NaCl. In general, the rejection increases with the valency of the solute; therefore, a higher rejection of multivalent (NH_4_)_2_SO_4_ than monovalent NaCl should be expected.

The values of the osmotic coefficient for (NH_4_)_2_SO_4_ decrease with an increase in the draw solution concentration from 0.96 at *c_f_* = 0.1 mol/L to 0.71 at *c_f_* = 0.5 mol/L. This decrease is consistent with the literature. For a similar concentration range, Guendouzi et al. reported a decrease in *ϕ* from 0.80 to 0.61 [44]. It is important to note that *ϕ* values in reference [44] were determined using the hygrometric method based on the relative humidity measurements above the solutions containing non-volatile electrolytes. Relative humidity is an example of a colligative property, and the resulting osmotic pressure and the corresponding osmotic coefficients are indirectly determined by experimental parameters. In contrast, *π* and *ϕ* values in Table 1 are the experimental parameters determined directly in the RO experiments.

**Figure 10:**
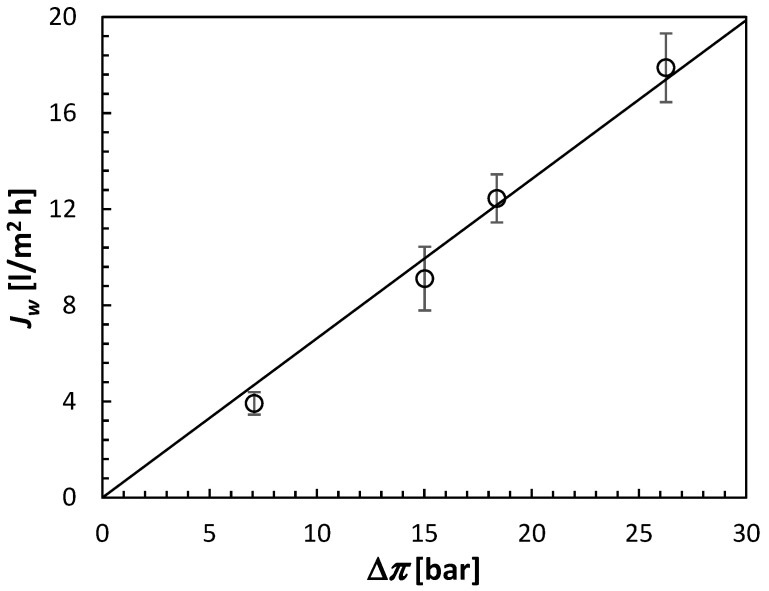
Water flux of the CTA membrane operated in the AL-FS orientation as a function of the corrected osmotic pressure of (NH_4_)_2_SO_4_ draw solution. All experiments were performed at 21.5 ± 0.5 °C using DI water as a feed solution.

The experimental osmotic pressures of (NH_4_)_2_SO_4_ from Table 1 are used to replot the water fluxes from the FO experiments in Figure. 10. It is evident that there is an excellent linear relationship between *J_w_* and Δ*π*, which passes through the origin. According to Equation (7), the slope in Figure 10 corresponds to the CTA membrane’s water permeability, A = 0.663 L/m^2^ h bar. This is significantly lower than the water permeability of the TFC used in the RO experiments. However, it is important to note that A of the TFC membrane is based on the water flux induced by hydraulic rather than osmotic pressure.

The next phase of this research will involve the actual NPK nutrients in a synthetic feed solution. With the NPK in the feed solution, the effective osmotic pressure gradient offered by the 0.5 mol/L (NH_4_)_2_SO_4_ would decrease, which might necessitate increasing the draw solution concentration. In turn, it would make the reconcentration of the draw solution in the subsequent RO process more challenging. At the same time, the current research indicated that hydraulic pressures as high as 40 bar do not lead to any significant compaction of the commercial PA-TFC membrane. Resolving the balance between the draw solution concentration and hydraulic pressure in the subsequent RO process, as well as determining the required membrane areas in both the FO and RO processes, would require preliminary design calculations. The experimental results with the NPK nutrients in the feed solution will enable these design calculations.

## 5. Conclusions

We have assessed the suitability of (NH_4_)_2_SO_4_ and NaCl as draw solutes for concentrating NPK nutrients in a series of FO experiments using a commercial CTA membrane and DI water as the feed solution. Although the water flux generated by NaCl was slightly higher than that by (NH_4_)_2_SO_4_, the reverse solute flux of the former was several times higher than that of the latter. Consequently, the CTA membrane exhibited a significantly greater selectivity for (NH_4_)_2_SO_4_ than for NaCl at any theoretical osmotic pressure. For example, at 36.6 bar, the selectivity for (NH_4_)_2_SO_4_ was 2400 compared to 565 for NaCl. In addition to the superior FO performance, ammonium sulfate contains nitrogen (i.e., one of the nutrients in the future liquid digestate). As such, a reverse solute flux (NH_4_)_2_SO_4_ would only increase the concentration of the N nutrient in the feed solution. Therefore, (NH_4_)_2_SO_4_ was chosen over NaCl for the second part of this project, in which we examined the reconcentration of (NH_4_)_2_SO_4_ in the RO experiments employing a commercial polyamide (PA) thin film composite (TFC) membrane, ACM4.

The RO experiments demonstrated the possibility of reconcentrating (NH_4_)_2_SO_4_ to 0.5 mol/L, with a corresponding water flux of 60 L/h m^2^ at 40 bar. In addition, the RO experiments at different feed concentrations and pressures enabled the experimental determination of the osmotic pressure of (NH_4_)_2_SO_4_ at various solute concentrations. The experimentally determined osmotic pressures were lower than those predicted from van’t Hoff’s equation. For example, the experimental osmotic pressure of (NH_4_)_2_SO_4_ at 0.5 mol/L was 26.3 bar compared to the theoretical value of 36.6 bar. The experimental osmotic pressures of (NH_4_)_2_SO_4_ reported in this work are consistent with those reported in the literature using an indirect hygrometric method. Moreover, the experimental osmotic pressures of (NH_4_)_2_SO_4_ correlated linearly with the FO water flux, further validating their correctness.

The results of this research lay the foundation for the next phase, which will involve using synthetic feed solutions containing NPK nutrients at concentrations similar to those in chemically pretreated liquid digestate. A 0.5 M (NH_4_)_2_SO_4_ will be employed as the draw solution. Forward osmosis (FO) tests with the CTA membranes will be conducted continuously over several days to measure the water extracted from the feed solution, ensuring the water flux—although it will decrease over time—remains within a reasonable range. Additionally, the diluted draw solution will be reconcentrated over several days in the RO system using the ACM4 membrane at 40 bars, while permeate is continuously removed to evaluate potential membrane compaction and concentration polarization effects. The findings from this next phase will support an initial economic analysis of the process, which will be further expanded in the final stage of this research involving different chemically pretreated liquid digestates.

## Figures and Tables

**Table 1:** Summary of the experimentally determined water permeability, osmotic pressure, solute permeability, and osmotic coefficient from RO experiments at 21.5 ± 0.5 °C and different feed pressures and concentrations of (NH_4_)_2_SO_4_.

*c_f_*	A	*π* (exp.)	*π* (Equation (3))	*ϕ* ^1^	B
[mol/L]	[L/m^2^ h bar]	[bar]	[bar]	[-]	[L/m^2^ h]
0.1	4.52	7.08	7.35	0.96	0.38
0.25	4.15	15.02	18.39	0.82	0.20
0.35	3.46	18.38	25.75	0.71	0.27
0.5	3.82	26.26	36.78	0.71	0.41

^1^ The ratio of experimental *π* and *π* from Equation (3).

## Data Availability

The data presented in this study are available on request from the corresponding author.

## Nomenclature

Symbols	
A	Water permeability coefficient [L/m^2^ h bar]
*A*	Area [m^2^]
B	Solute permeability coefficient [L/m^2^ h]
*c*	Molar concentration [mol/L]
*i*	The number of ions when the solute dissociates [-]
*J*	Flux [L/m^2^ h or kg/m^2^ h]
*m*	mass [kg or g or mg]
*L*	Membrane thickness [m]
*p*	Pressure [bar]
*R*	Universal gas constant [J/mol K]
R	Solute rejection [-]
S	FO selectivity [L/kg]
*T*	Absolute temperature [K]
*t*	Time [s]
*V*	Volume [L]
Greek symbols	
Δ*c*	Molar concentration gradient [mol/L]
Δ*p*	Hydraulic pressure gradient [bar]
Δ*π*	Osmotic pressure gradient [bar]
Δ*t*	Elapsed time [h]
Δ*V*	Collected volume
*ϕ*	Osmotic coefficient [-]
*π*	Osmotic pressure [bar]
*ρ*	Density [kg/m^3^]
Subscripts	
*d*	Draw solution
*f*	Feed solution
*FS*	Feed solution
*m*	Membrane
*p*	Permeate solution
*s*	Solute
*w*	Water
